# Adopting Global Lung Function Initiative Reference Equations Alters Pulmonary Function Testing Interpretation in a Safety-Net Cohort

**DOI:** 10.2174/0118743064497297260820041654

**Published:** 2026-08-24

**Authors:** Stephen T. Russell, Jenny E. Han, Jordan A. Kempker, Eric Honig, Abiodun T. Kukoyi

**Affiliations:** 1 Division of Pulmonary, Allergy, Critical Care, and Sleep Medicine, Emory University School of Medicine, Atlanta, GA, USA; 2 Grady Health System, Atlanta, GA, USA

**Keywords:** Spirometry, Lung volumes, Diffusing capacity, Racial disparities, Respiratory function tests, Global lung initiative, Reference ranges

## Abstract

**Introduction/Objective:**

Current American Thoracic Society/European Respiratory Society (ATS/ERS) guidelines recommend Global Lung Initiative (GLI) reference equations for spirometry, lung volumes, and diffusing capacity (DLCO). The impact of adopting these reference equations remains unclear and may differ by race.

**Methods:**

We conducted a cross-sectional study of Pulmonary Function Tests (PFT) performed at Grady Memorial Hospital in Atlanta, GA, from 2017 to 2024. PFT components and interpretive patterns were analyzed using current Grady reference equations, then compared with GLI, including race-neutral spirometry, and stratified by race.

**Results:**

There were 7877 eligible patients; 7034 (89%) were Black, 529 (7%) were White, and 314 (4%) were Hispanic. Black patients had increased frequency of abnormal FEV1 (37.4% to 52.1%, *p* < 0.001), FVC (32.4% to 48.4%, *p* < 0.001), Total Lung Capacity (TLC) (6.7% to 40.8%, *p* < 0.001), and DLCO (30.3% to 45.3%, *p* < 0.001). White and Hispanic patients had decreases in abnormal FEV1 (48.0% to 38.0%, *p* < 0.001, and 29.0% to 20.7%, *p* < 0.001, respectively), decreases in abnormal FVC (38.0% to 26.1%, *p*< 0.001, and 26.1% to 18.2%, *p* < 0.001), increases in abnormal TLC (12.7% to 17.8%, *p* < 0.001, and 12.4% to 16.8%, *p* < 0.001) and no change in DLCO. 44.3% of Black, 16.8% of White, and 14.5% of Hispanic patients had a new interpretive pattern.

**Discussion:**

In this health system with a predominantly Black patient population, transitioning our PFT reference equations to GLI increased detection of abnormalities and changed interpretive patterns, with greater effects in Black patients.

**Conclusion:**

The health-system impact of implementing GLI reference equations is highly context-dependent, influenced by demographics and prior reference equations.

## 1 INTRODUCTION

Pulmonary Function Testing (PFT) assists in identifying respiratory-related physiologic abnormalities by comparing an individual’s performance to a reference standard of healthy individuals for a given age, height, sex, and, controversially, race. The American Thoracic Society/European Respiratory Society (ATS/ERS) Interpretive Strategies for Pulmonary Function Testing recommends adopting the race-neutral Global Lung Function Initiative (GLI-global) reference equations for spirometry, and GLI equations for lung volumes and diffusing capacity [1-3]. Numerous studies have shown that the adoption of GLI-global, compared to GLI-race-specific spirometry equations, leads to more spirometry readings categorized as abnormal in Black patients and fewer in White patients [3-12]. Further, there is growing evidence that GLI-global is equivalent to, or better than, race-specific equations for predicting important patient-related outcomes such as breathlessness, six-minute walk distance, quality of life, and mortality in secondary analyses of existing cohort studies [4, 5, 13-15]. However, most of these studies have focused on spirometry and therefore the effects on the interpretation of Total Lung Capacity (TLC) and DL_CO_ are understudied. Unique to these specific portions of the PFT, the GLI reference equations for TLC and DL_CO_ were developed for White populations, and the effects of applying these ranges to a racially diverse population merit further investigation [16]. Within this context, our study uses a large, predominantly Black cohort from an urban safety-net hospital to evaluate the frequency and pattern of PFT reclassification when applying GLI global reference ranges, stratified by race.

## 2 METHODS

### 2.1 Study Design and Setting

We performed a cross-sectional study of 7,877 sequential PFTs completed at Grady Memorial Hospital, Atlanta, GA, between 2017 and 2024. We excluded patients with unknown race and those of Asian descent due to a small sample size. If patients had multiple tests, only the first was included. Reporting of this observational study follows the STROBE guidelines. A completed STROBE checklist is provided in the Supplementary Material. This study was conducted in accordance with the ethical standards of the Declaration of Helsinki. This study was approved by the Emory IRB (IRB #00006312).

### 2.2 Pulmonary Function Testing Components

All spirometry, static lung volume by plethysmography, and diffusing capacity measurements were obtained using MedGraphic instrumentation and software (MGC Diagnostics, St. Paul, MN). The PFT laboratory adheres to ATS technical standards and performs routine quality assurance and equipment calibration. From each PFT, we obtained the raw pre-bronchodilator Forced Expiratory Volume in 1 second (FEV_1_), Forced Vital Capacity (FVC), FEV_1_/FVC ratio, Total Lung Capacity (TLC), and Diffusing Capacity (DL_CO_). Race was either patient-reported or technician-assigned.

### 2.3 PFT Reference Equations

During the study period, the PFT lab used the regression equations described in Table **1** to determine a Lower Limit of Normal (LLN). Prior to the 2021 ATS/ERS guidelines, there were no established recommendations for lung volume and DL_CO_ reference equations. When referring to overall interpretive patterns, which incorporate spirometry, lung volumes, and DL_CO_, we will refer to the reference equations in sum as ‘Grady’ or ‘GLI’ equations for ease of reading. To determine the LLN by the GLI reference equations, we entered each patient’s raw PFT values, along with their height, age, and sex, into the online GLI calculator (https://gli-calculator.ersnet. org/). We used the GLI-global equation for spirometry.

### 2.4 PFT Patterns

Each PFT component (FEV_1_, FVC, FEV_1_/FVC, TLC, and DL_CO_) was determined to be normal or abnormal using Grady reference equations and then using GLI equations. Abnormal was defined as the raw value being less than the LLN. Normal was defined as the raw value greater than or equal to the LLN. After evaluating the PFT components in isolation, each patient was assigned an interpretive pattern using both sets of reference equations, in accordance with ATS guidelines for the interpretation strategies of pulmonary function testing. The PFT patterns were: normal, obstruction, restriction, mixed, non-specific pattern, and isolated low DL_CO_ (Table **2**).

### 2.5 Statistical Analyses

Changes in frequency of abnormal PFT components and PFT patterns is reported in absolute numbers and percentages in **Supplementary Tables 1** and **2**, respec-tively. Differences between the proportion of normal and abnormal test results in each PFT component by reference range equation were evaluated using McNemar’s test for paired binary data. A two-sided *p*-value < 0.05 was considered statistically significant. Continuous variables were described using means and standard deviations for normally distributed continuous variables and percentages for categorical variables. All statistical analysis was performed using SAS 9.4 (Cary, NC).

## 3 RESULTS

### 3.1 Patient Characteristics

Our cohort comprised 10,213 studies conducted between 2017 and 2024. After exclusions, 7,877 unique patients' studies remained for analysis (Fig. **1**). Of included patients, 7,034 (89.3%) were Black, 529 (6.7%) were White, and 314 (4.0.%) were Hispanic. The mean age was 57 years (standard deviation (SD):12.7), and 4907 (62.3%) patients were female. Table **3** displays patient characteristics including raw PFT values, stratified by race and ethnicity.

### 3.2 PFT Component Reclassification when Applying GLI Reference Ranges

#### 3.2.1 Spirometry

Among Black patients, our analyses follow the PFT lab’s transition from GLI-race specific to GLI-global. Regarding FEV_1_, 1031 (14.7%) studies were reclassified from normal to abnormal while zero were reclassified from abnormal to normal; (**Supplementary Table 3**). Regarding FVC, 1124 (16.0%) studies were reclassified from normal to abnormal while zero were reclassified from abnormal to normal. Regarding FEV_1_/FVC, there was minimal reclassification (<1%) in either direction (**Supplementary Table 1**).

Among White patients, our analyses follow the PFT lab’s transition from GLI race-specific to GLI-Global spirometry equations. Regarding FEV_1_, no studies were reclassified from normal to abnormal, while 53 (10.0%) were reclassified from abnormal to normal. Regarding FVC, 1 (0.2%) study was reclassified from normal to abnormal, while 64 (12.1%) were reclassified from abnormal to normal. Regarding FEV_1_/FVC, there was a minimal change (< 1%) in reclassification in either direction.

Among Hispanic patients, our analyses follow the PFT lab’s transition from GLI race-specific to GLI-Global spirometry equations. Regarding FEV_1_, no studies were reclassified from normal to abnormal, while 26 (8.3%) were reclassified from abnormal to normal. Regarding FVC, no studies were reclassified from normal to abnormal, while 25 (8%) were reclassified from abnormal to normal. Regarding FEV_1_/FVC, there was no change in classification in either direction.

#### 3.2.2 Lung Volumes

Our analyses follow the PFT lab’s transition from Boren to GLI equations. Regarding TLC among Black patients, 2358 (34.2%) studies were reclassified from normal to abnormal, and 2 (0.03%) studies were reclassified from abnormal to normal. Among White patients, 28 (5.4%) of studies were reclassified from normal to abnormal and 1 (0.2%) study was reclassified from abnormal to normal. Among Hispanic patients, 15 (4.8%) of studies were reclassified from normal to abnormal and none were reclassified from abnormal to normal (Fig. **2** and **Supplementary Table 4**).

#### 3.2.3 Diffusing Capacity

Our analyses follow the PFT lab’s transition from NHANES I to GLI reference equations. Regarding DL_CO_ among Black patients, 1146 (16.4%) of studies were reclassified from normal to abnormal and 89 (1.3%) were reclassified from abnormal to normal. Among White patients, 31 (5.9%) of studies were reclassified from normal to abnormal and 21 (4.0%) were reclassified from abnormal to normal. Among Hispanic patients, 7 (2.2%) of studies were reclassified from normal to abnormal and 9 (2.9%) were reclassified from abnormal to normal; (Fig. **2** and **Supplementary Table 5**).

#### 3.2.4 Changes in Overall Interpretative Patterns

Our analyses follow the PFT lab’s transition from Grady to GLI reference equations. Among Black patients, the percentage of studies with a normal pattern decreased from 44.6% to 24.2%. Decreases were observed in obstruction (19.7% to 15.6%), non-specific pattern (22.0% to 13.7%), and isolated low DL_CO_ (7.1% to 5.7%). Increases were observed in restriction (6.1% to 36.6%) and mixed obstruction/restriction (0.5% to 4.3%).

Among White patients, the percentage of studies with a normal pattern increased from 37.7% to 43.0%. Decreases were observed in the non-specific pattern (19.5% to 8.6%). Increases were observed in restriction (11.9% to 16.6%). There were < 1% changes in obstruction, mixed obstruction/restriction, and isolated low DL_CO_.

Among Hispanic patients, the percentage of studies with a normal pattern increased from 61.3% to 65.5%. Decreases were observed in non-specific pattern (16.5% to 7.7%) and isolated low DL_CO_ (3.9% to 2.6%). Increases were observed in restriction (11.9% to 16.5%). There were < 1% changes in obstruction and mixed obstruction/ restriction. Raw data is shown in **Supplement****ary** **Table 2**, and Fig. (**3**) presents a Sankey diagram of individual patient changes.

## 4 DISCUSSION

In a diverse clinical pulmonary function testing lab, we reanalyzed full pulmonary function test results using GLI reference ranges to assess the frequency and pattern of reclassification by race and ethnicity. Black patients had greater increases in abnormalities across all PFT domains compared with White and Hispanic patients. Using GLI equations, Black patients had a decrease in the frequency of a normal study, whereas White and Hispanic patients had an increase in the frequency of a normal study. Changes in interpretive pattern were observed more frequently in Black patients.

This study has several limitations. First, the impact of adopting a new reference equation depends on the previous reference equations used. Previous ATS/ERS guidelines did not specify preferred reference equations for lung volumes and DL_CO_, and there may be significant variability in reference equations among PFT labs. Therefore, our results for lung volumes and DL_CO,_ as well as our observed shifts in interpretive patterns, may not be generalizable to other PFT laboratories and patient cohorts. Second, race was either self-reported or inferred by the technician. This method does not consider the genetic and ancestral diversity that may affect reclassification. Specifically, our cohort's Black patients (about 90%) include an unspecified number of immigrants from Africa and the Caribbean, as well as Black American patients whose ancestors have lived in the United States for multiple generations. We could not account for these differences in heritage, so their impact on reclassification is unknown. Third, although PFT were conducted according to current ATS/ERS standards, we included all unique PFT in our analysis, including studies of different quality grades. Fourth, our analyses show only direction and magnitude of changes in PFT interpretive patterns and do not necessarily infer improved detection of respiratory conditions. Further work is needed to validate the sensitivity and specificity of the GLI reference equation in diverse populations, especially for lung volumes and diffusing capacity. Fifth, the changes in PFT interpretation are at least in part due to our existing reference equations and predominantly Black patient population, which limit external validity for other PFT labs with different equations and patient populations. We hope these new findings in a predominantly Black population can help address the gap in diverse research and encourage broader inclusion of other patient populations in future studies.

When focusing on spirometry, the comparison of PFT interpretations using GLI race-specific and race-neutral equations showed that the direction and magnitude of reclassification by race were consistent with previous studies (8, 10, 13). We found an absolute increase in abnormal FEV_1_ and FVC in Black patients and decreases in White and Hispanic patients. There were minimal changes in the FEV_1_/FVC ratio across all races, also consistent with previous studies [10]. Novel to this study are the findings of a decrease in the frequency of the non-specific pattern in White and Hispanic patients when the GLI global reference range is applied, as well as the impact on overall PFT interpretation after adopting the GLI reference equations.

The current ATS/ERS guidelines are the first set of guidelines to specify a preference for reference equations for lung volumes and D_LCO_. It is important to note that the recommended GLI-reference equations are based on White/Caucasian populations only; hence, unlike the GLI-Global equations, they are not race-agnostic [17, 18]. Applying GLI reference ranges resulted in larger percentages of abnormalities in Black patients in our cohort, though it is also notable that rates of TLC and DL_CO_ abnormalities increased in White and Hispanic patients as well, but by a smaller magnitude. This is in contrast to a study comparing GLI and ECCS equations in White patients that found a decrease in abnormalities when using GLI reference equations [19]. Other studies have also suggested that changes in DL_CO_ abnormalities depend on the baseline reference equation used [20-22]. Thus, while the rates of reclassification will likely depend on each PFT lab’s existing reference ranges and patient populations, centers that serve larger Black populations are likely to see a relatively large increase in abnormal PFT studies at the time of transition to GLI equations.

As already highlighted, the large increase in the restrictive pattern that we found may be due in part to our existing reference ranges. The comparison reference range for males, Boren, dates to 1964 and had a high rate of tobacco use in the “healthy” subjects from which it was derived [23]. Our lab’s use of a 12% correction for Black patients’ lung volumes also likely contributed to a very low number of Black patients’ pre-analysis PFT showing restriction. Taken together, these two factors – the initial reference equations used and the race correction - amplified the magnitude of reclassification from normal to abnormal lung volumes in Black patients.

While much work and attention have focused on spirometry, the absence of race-neutral or race-composite reference equations for lung volumes and DL_CO_ raises concerns that inequities remain in the interpretation of pulmonary function testing. Further research is needed to assess the sensitivity and specificity of the currently recommended GLI equations for detecting disease, especially in non-White cohorts that were under-represented in their derivation. Our analysis shows altered PFT interpretive patterns in 41% of cases, which may have downstream effects on diagnosis, treatment, and additional testing for affected patients.

Although the effects of adopting GLI equations will depend on a local PFT lab’s existing reference ranges and the racial makeup of the population, there are likely to be some differences in PFT interpretations that result from changing reference ranges. While it is not likely logistically feasible to alter previously completed PFTs, it will be important for PFT laboratories to prospectively communicate changes in reference ranges so that clinicians can interpret individual studies and longitudinal trends within that context [24]. In our laboratory, we included a message in PFT reports indicating that recent changes in reference ranges may affect PFT inter-pretation, alerting the providers interpreting the report as well as other providers using the PFT results for clinical decision-making.

While the debate over the effects of race-neutral *versus* race-specific spirometry is complex, adding lung volumes and DL_CO_ only further complicates the under-standing of the impacts of reference equation changes at the individual, institutional, and national levels.

## CONCLUSION

In a majority Black cohort, adoption of GLI reference ranges increased the proportion of PFT studies with at least one abnormality by 18%, with nearly 75% of studies now showing an abnormality (an average of 200 studies/year analyzed). This effect was most pronounced in Black patients compared with White or Hispanic patients. Over 40% of studies had a different interpretive pattern with GLI reference equations compared with the previously used reference equations. The impact of adopting the GLI equations will vary based on the demographic composition of each healthcare system and the existing reference ranges in use.

## Figures and Tables

**Fig. (1):**
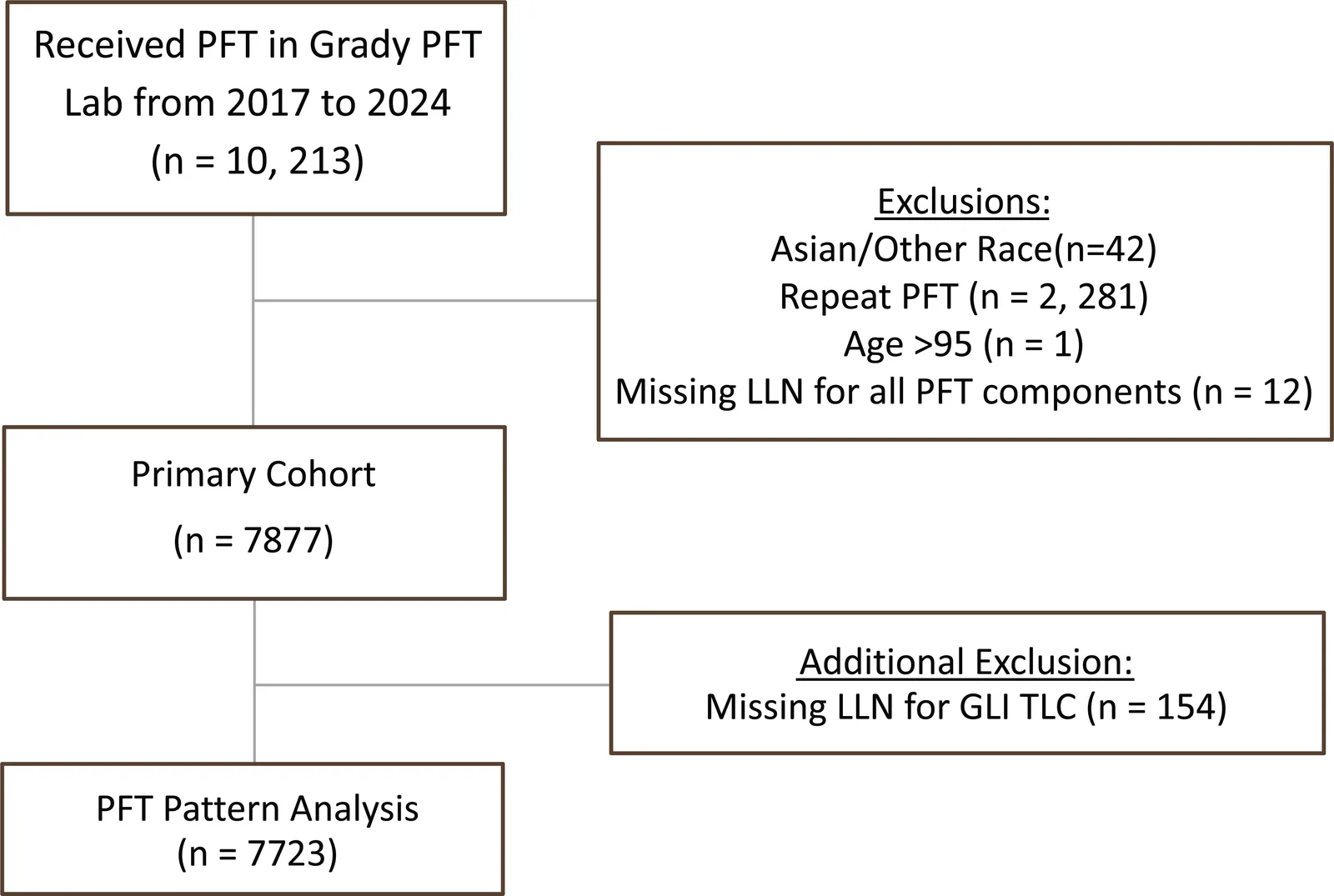
Study flow diagram. **Abbreviations:** PFT = Pulmonary Function Test. Grady = Grady Memorial Hospital, Atlanta, GA. Pattern Analysis = Assessment for changes in overall PFT pattern after analyzing with GLI reference equations. LLN = Lower Limit of Normal, TLC=Total Lung Capacity.

**Fig. (2):**
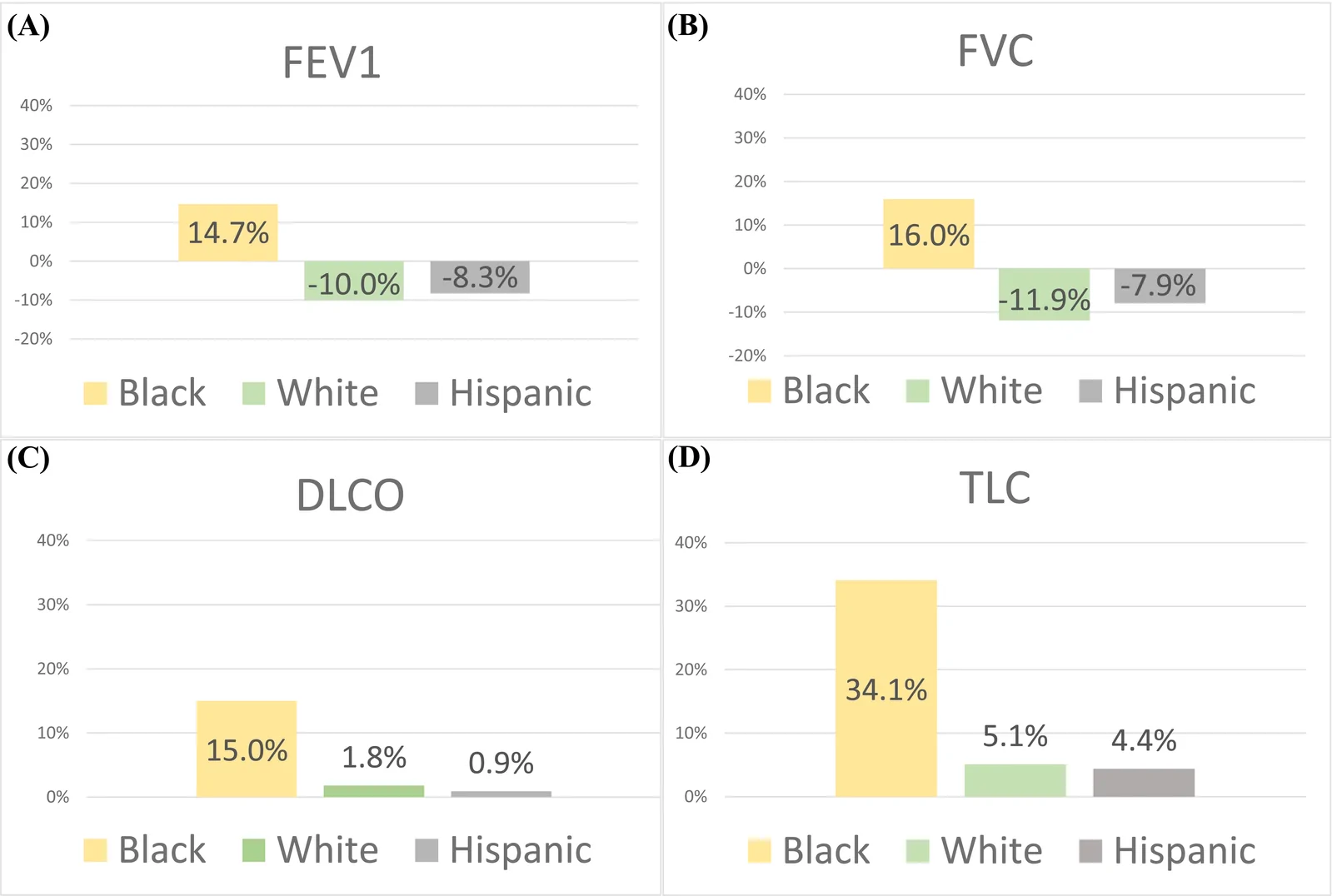
Absolute increases and decreases in abnormal PFT component comparing grady and GLI reference equations, stratified by race. Bar graphs illustrating the magnitude and direction of reclassification by race for FEV_1_ (Panel A), FVC (Panel B), TLC (Panel C), and DL_CO_ (Panel D) when these parameters were reinterpreted using a GLI reference equation. Percentages are net absolute differences. Percentage values greater than 0 indicate increases in abnormal values obtained (*i.e*., reclassification from normal to abnormal). Percentage values less than 0 indicate a decrease in the number of abnormal values (*i.e*., reclassification from abnormal to normal). For spirometry, the comparison reference ranges were GLI-race specific to GLI-race neutral. For lung volumes, the comparison reference ranges were Boren and GLI. For DL_CO_, the comparison reference ranges were NHANES I and GLI.

**Fig. (3):**
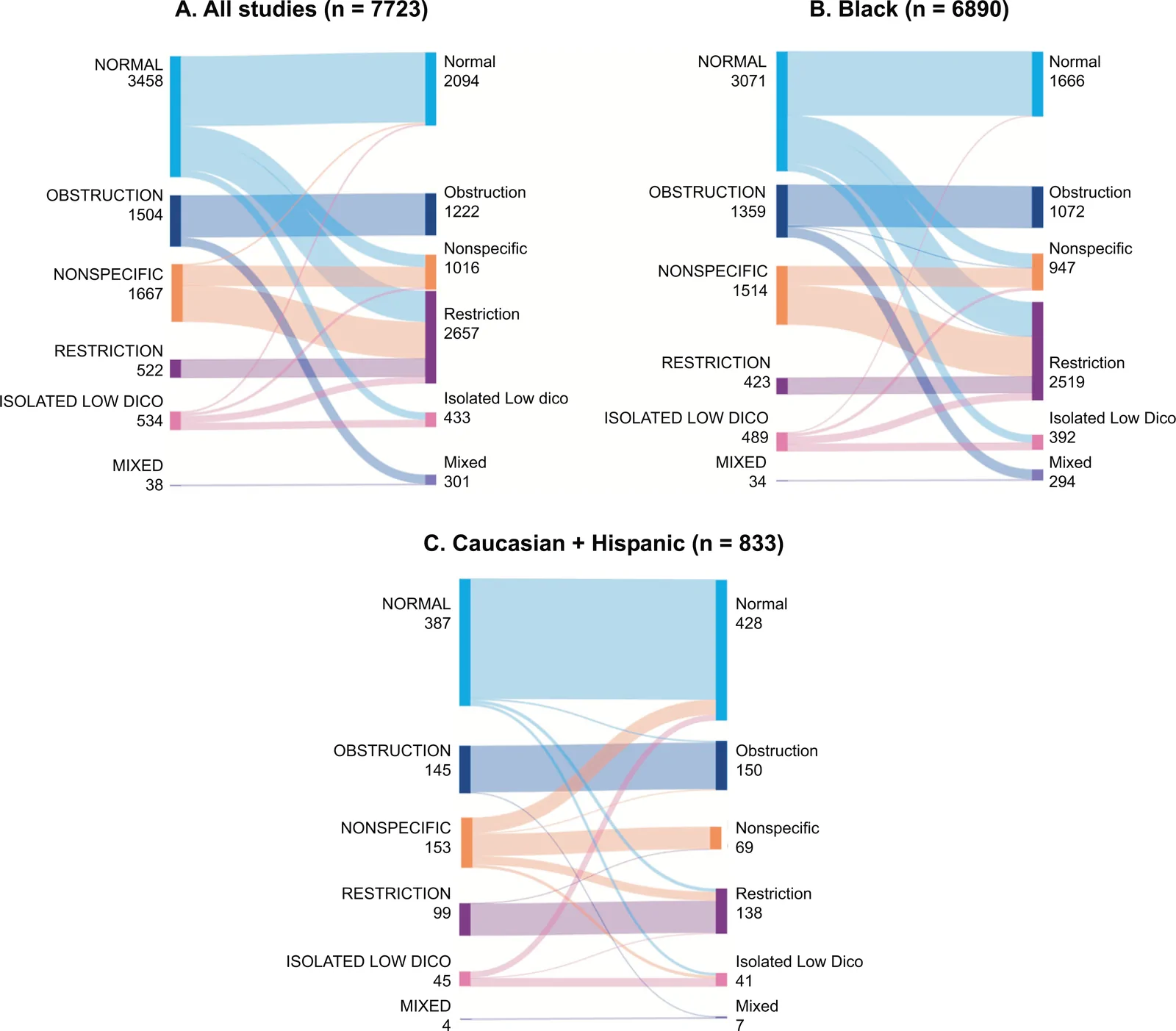
Overall changes in interpretive patterns when applying GLI Reference equations, stratified by race. Alluvial plots demonstrating changes in the overall interpretation of pulmonary function studies from the entire cohort (**A**), Black (**B**), and combined White and Hispanic (**C**) individuals as having normal lung function, obstructive, nonspecific, restrictive, mixed obstruction/restriction, and isolated diffusion abnormalities. Flows between axes represent changes in interpretation between the two sets of reference equations. For spirometry, the comparison reference ranges were GLI-race specific to GLI-race neutral. For lung volumes, the comparison reference ranges were Boren and GLI. For DL_CO_, the comparison reference ranges were NHANES I and GLI. **Abbreviations:** DL_CO_: Diffusing Capacity.

**Table 1:** Grady PFT reference equations.

**PFT Component**	**Race**		
-	Black	White	Hispanic
Spirometry	GLI-Black	GLI-Caucasian	
TLC	Boren (M)/ECCS (F)*		
DL_CO_	NHANES-1^$^		

**Abbreviations:** TLC: Total Lung Capacity, DL_CO_: Diffusing Capacity, NHANES: National Health and Nutrition Examination Survey, ECCS: European Community of Coal and Steel, GLI: Global Lung Initiative. ^*^Race correction 0.88 for TLC applied to Black patients. ^$^Equation applied to Black patients differs from that applied to White or Hispanic patients. However, they are derived from NHANES-I.

**Table 2:** PFT interpretation patterns.

**Pattern**	**Definition**
Obstruction	FEV_1_/FVC<LLN and TLC>LLN
Restriction	FEV_1_/FVC>LLN and TLC<LLN
Mixed Obstructive and Restriction	FEV_1_/FVC<LLN and TLC<LLN
Non-Specific Pattern	FEV_1_ and/or FVC<LLN and FEV_1_/FVC>LLN and TLC>LLN
Isolated Low Diffusing Capacity	FEV_1_, FVC, FEV_1_/FVC, and TLC>LLN, DL_CO_ <LLN

**Abbreviations:** PFT: Pulmonary Function Test, FEV_1_: Forced Expiratory Volume in 1 Second, FVC: Forced Vital Capacity, TLC: Total Lung Capacity, DL_CO_: Diffusing Capacity, LLN: Lower Limit of Normal.

**Table 3:** Patient characteristics.

**Characteristic**	**Black** **(n = 7034)**	**White** **(n = 529)**	**Hispanic** **(n = 314)**	**Overall** **(n = 7877)**
Age, years, mean (SD)	57.3 (12.7)	56.8 (11.6)	50.6 (13.1)	57.0 (12.7)
Female Sex, n (%)	4475 (63.6)	221 (41.8)	211 (67.2)	4907 (62.3)
BMI (kg/m^2^) mean (SD)	31.9 (9.5)	30.5 (9.2)	30.6 (7.3)	31.8 (9.4)
Raw Pre-BronchodilatorPFT Values, L, mean (SD)	-	-	-	-
FEV_1_	1.99 (0.68)	2.43 (0.88)	2.42 (0.73)	2.04 (0.71)
FVC	2.64 (0.84)	3.38 (1.04)	3.00 (0.90)	2.70 (0.88)
FEV_1_/FVC	76.11 (12.68)	72.12 (13.95)	81.28 (9.40)	76.05 (12.74)
TLC	4.91 (1.15)	6.05 (1.40)	5.02 (1.14)	4.99 (1.20)
DL_CO_	18.41 (7.79)	22.37 (8.69)	23.33 (8.82)	18.87 (0.90)

**Abbreviations:** FEV_1_: forced Expiratory Volume in 1 Second, FVC: Forced Vital Capacity, TLC: Total Lung Capacity, DL_CO_: Diffusing Capacity, BMI: Body Mass Index, SD: Standard Deviation.

## Data Availability

The datasets generated or analyzed during the current study are not publicly available due to IRB-imposed limitations. Data may be available in part from the corresponding author [S.T.R] on special request.

## LIST OF ABBREVIATIONS

ATS: American Thoracic Society
ERS: European Respiratory Society
GLI: Global Lung Function Initiative
DLCO: Diffusing Capacity
PFT: Pulmonary Function Test
TLC: Total Lung Capacity
FEV1: Forced Expiratory Volume in 1 Second
FVC: Forced Vital Capacity
LLN: Lower Limit of Normal
SD: Standard Deviation

## SUPPLEMENTARY MATERIAL

Supplementary material is available on the publisher’s website along with the published article.
